# Cholera in Chicago

**Published:** 1850-09

**Authors:** 


### ARTICLE V.

### CHOLERA IN CHICAGO.

We clip from the *Daily Journal* the following statement of the total mortality, presuming that it is as nearly as possible correct:—

" The total number of deaths in July, 240 ; total in August, 466.

Deaths from Cholera from the 23d of June, the time when the epidemic made its appearance, to the first of August, 158 ; from the 1st of Aug. to 1st of Sept., 283, making a total of deaths by Cholera during the season, of 441.

Thus the whole mortality of the city, from the 23d of June to the 1st. of September, a period of 69 days, is not far from 624, making an average of about nine per day, which, taking the population at 27,000, gives one death daily to every 3,000."

We hope hereafter to present a fuller report of the subject. The disease has now (Sept. 1st) almost totally disappeared.

